# Reproducibility and Validity of a Food Frequency Questionnaire Designed to Assess Diet in Children Aged 4-5 Years

**DOI:** 10.1371/journal.pone.0167338

**Published:** 2016-11-29

**Authors:** Jesus Vioque, Daniel Gimenez-Monzo, Eva Maria Navarrete-Muñoz, Manuela Garcia-de-la-Hera, Sandra Gonzalez-Palacios, Marisa Rebagliato, Ferran Ballester, Mario Murcia, Carmen Iñiguez, Fernando Granado, INMA-Valencia Cohort Study

**Affiliations:** 1 Unidad de Epidemiologia de la Nutrición. Universidad Miguel Hernández, Alicante, Spain. Institute for Health and Biomedical Research (ISABIAL—FISABIO Foundation), Alicante, Spain; 2 CIBER de Epidemiología y Salud Pública (CIBERESP), Madrid, Spain; 3 Foundation for the Promotion of Health and Biomedical Research in the Valencian Region (FISABIO/CSISP), Valencia, Spain; 4 Departamento de Medicina, Universitat Jaume I, Castellón de la Plana, Spain; 5 Servicio Bioquímica Clínica, Hospital Universitario Puerta de Hierro-Majadahonda, Madrid, Spain; Universidade de Sao Paulo, BRAZIL

## Abstract

**Background:**

The food frequency questionnaire (FFQ) is the most efficient and cost-effective method to investigate the relationship between usual diet and disease in epidemiologic studies. Although FFQs have been validated in many adult populations worldwide, the number of valid FFQ in preschool children is very scarce. The aim of this study was to evaluate the reproducibility and validity of a semi-quantitative FFQ designed for children aged 4 to 5 years.

**Materials and methods:**

In this study, we have included 169 children aged 4–5 years from the INMA project in Valencia, a population-based prospective cohort study of mothers and children in Spain. The 105-items FFQ was administered twice to the parents or care-givers of children over a 9-month period. Reproducibility was explored by comparing intake of nutrients by the FFQs, while validity was examined by comparing the nutrient values from the FFQs with the average nutrient values of three 24 hour dietary recall (24hDR) taken in the period, and also, with the concentration in blood specimens for several vitamins (carotenoids, folate, vitamin B12, vitamin C and α-tocopherol). Pearson correlation coefficients and de-attenuated correlation coefficients were calculated and we also evaluated misclassification by quintile distribution.

**Results:**

All correlation coefficients for reproducibility for nutrients and major food groups were statistically significant; the average correlation coefficients for daily intake were 0.43 for food groups and 0.41 for nutrients. The average correlation coefficients for validity for daily intakes against 24hDR was r = 0.30, and the average for de-attenuated correlation coefficients was r = 0.44. When evaluating validity against the blood concentration of vitamins, statistically significant correlations were observed for vitamin C (0.35), lycopene (0.31), β-Cryptoxantin (0.40), and vitamin E (0.29); the average of correlation coefficients was r = 0.21.

**Conclusion:**

Despite some low to moderate correlations for reproducibility and validity, overall this study suggests that the FFQ may be a good method for assessing a wide range of food groups and nutrients intake in children aged 4–5 years.

## Introduction

The importance of early nutrition for child health and long-term health has been highlighted over the last two decades and reaffirmed more recently [1–3]. However, young children’s diet is a complex exposure that is difficult to measure and requires valid dietary assessment methods in order to explore relationships with health outcomes.

At present, food frequency questionnaires (FFQ) are the preferred dietary assessment method in most epidemiologic studies mainly due to their low cost and ease of administration. However, all self-reporting methods of food intake are subject to errors, and therefore, validation studies are necessary to assess the effect of measurement error and to avoid as far as possible incorrect estimations [4]. Thus, a few hundred FFQs have probably been validated in different populations around the world, mostly in adult populations [5,6] and to a lesser extent in adolescents [7]. Nonetheless, the number of validity studies of FFQs to assess food and nutrient intakes in young children is much lower, probably as a result of the many dietary changes at these ages due to the rapid development and growth of the child [4]. In a systematic review of thirty-two articles by Ortiz-Andrellucchi on dietary assessment methods for micronutrient intake in infants, children and adolescent, eight studies presented validation data for FFQ in preschool children aged 2–5 years [8], although only two studies showed that past dietary intake of preschool children could be measured reasonably well when comparing their results with food records or dietary recalls [9, 10]. Only one study showed a good correlation for vitamin C and weak or no correlation for other micronutrients (vitamin D, retinol and b-carotene), when dietary intakes from FFQ were compared with plasma concentrations [11].

More recently published studies have shown that FFQ may produce valid and satisfactory estimates of a wide range of food and nutrient intakes in preschool children of Greece [12], Lebanon [13] and Denmark [14]. However, there is clearly a need for more valid FFQs in preschool children in many countries including Spain, where, as far as we know, no valid FFQ exists for dietary assessment of food and nutrients in children aged 4 to 5 years. Regarding the reference methods to validate FFQ, the most frequently used, have been food records and 24 hour dietary recall (24hDR), although food records have been used less because they are more demanding and require a high level of motivation to be completed [15]. When available, biomarkers may be an alternative or supplementary reference method for the validation of some nutrient intakes since their measurement errors are independent of those of FFQ [16].

The aim of this study was to evaluate the reproducibility and validity of a semi-quantitative FFQ designed to assess the diet of children aged 4 to 5 years, against three 24hDR and several nutrient biomarkers, in the INMA Project [17], a prospective cohort study of mothers and children in Spain.

## Material and Methods

### Study population

Subjects in this study were 169 healthy children aged 4–5 years enrolled in the Spanish Childhood and Environment Project of Valencia (the INMA study), a multicenter mother-children prospective cohort study designed to investigate the effect of environmental exposures and diet during pregnancy and childhood [17]. Participant recruitment and follow-up procedures in the Valencia cohort have been reported in detail elsewhere [18,19]. Briefly, pregnant women who agreed to participate in Valencia gave birth to 787 singleton live infants between May 2004 and February 2006. At the age of 4–5 years, 590 children attended the planned follow-up visit when approximately one out of three parents were invited to participate in the validation study. Final analyses included 169 children whose parents agreed to participate, most of whom (n = 165) also provided blood samples at baseline; this sample size was estimated to guarantee statistical significance for correlation coefficients r ≥0.20 which may still be of interest in validity studies [6]. Flowchart is shown in Supporting Information (S1 Fig). All parents provided written informed consent. The Ethics Committees of the La Fe Hospital in Valencia and Miguel Hernández University approved the research protocol.

Trained nutritionists carried out personal interviews with the parents to obtain extensive information on child characteristics, and physical examination to measure the weight and height of the children following standard protocols. Body mass index (BMI) was calculated by dividing the measured weight in kg by the square of the measured height in meters, and further classified following the Cole criteria [20]. Questionnaires were used to obtain information on the socioeconomic status of the parents according to the Spanish adaptation of the British classification system (three categories: I/II [high]; III; and IV/V [low]), educational level (primary/none; secondary; and university), country of origin (Spain or other), and the use of preschool canteens (less than once per week; once or more times per week).

### Dietary assessment: semi-quantitative Food Frequency Questionnaire

A semi-quantitative FFQ of 105 food items was used to assess the usual daily intake of foods and nutrients (available at: http://bibliodieta.umh.es/files/2011/07/CFA105.pdf). The FFQ was derived from an adult version of the FFQ that had previously been validated among the mothers of the children [21]. The adult FFQ had a similar structure to the Harvard questionnaire [22], and was modified to include food items and portion sizes appropriate for children aged 4 to 5 years. A discussion group was created, involving two nutritional epidemiologists and two dietitian-nutritionist, to remove several items that children were unlikely to report (e.g., offal, tripe, alcoholic drinks) whereas we expanded the list of food items for dairy products, sweets and sugary foods. In addition, the group agreed to use in the FFQ, small portion sizes for specific food items based on nutritional survey carried out in Spanish children aged 6–9 years and other published sources for this age group [23–27]

The range of the coefficients of reproducibility and biochemical validity of the adult FFQ was similar to that observed in other established diet questionnaires in the literature [5].

Parents were asked twice, at baseline and approximately 9 months later, to report how often, on average, their children had consumed the specified serving or portion size for each food item of the FFQ in the previous year. The questionnaire had nine possible responses, ranging from ‘never or less than once per month’ to ‘six or more per day’.

Nutrient values were primarily obtained from the food composition tables of the US Department of Agriculture publications as well as other published sources for specific Spanish foods and portion sizes [27–29]. In order to obtain average daily nutrient intakes from diet for each child, we multiplied the frequency of use for each food by the nutrient composition of the portion/serving size specified on the FFQ and added the results across all foods. As the percentage of children taking supplements was very low (<2%), the total daily nutrient intake was based only on the estimates from the FFQ. We also estimated the mean daily consumption for 17 foods and food groups by grouping the intake of specific foods in the FFQ (Table 1).

**Table 1:** Specific food items included in the analysis of food groups.

Foods/ Food groups (#of foods)	Foods
**Dairy Products (14)**	whole milk; semi-skimmed milk fortified milk; pre or probiotic yogurt; whole and low fat yogurt; whole and low-fat cheese; flavoured milk; Petit Suisse; custard, cream caramel, pudding; ice-cream
**Eggs (1)**	Eggs
**White meat (2)**	chicken or turkey with; and without skin;
**Red meat (4)**	beef, pork or lamb; liver; offal; hamburger
**Processed meat (4)**	ham, salami and others; sausages; pate; bacon
**Fatty fish (5)**	swordfish, bonito, and fresh tuna; small oily fish (mackerel, sardine; anchovy); canned tuna; canned sardine or mackerel; dry or smoked fish.
**Lean fish (2)**	hake, sole, gilthead and similar white fish type; assorted or mixed fried fish
**Seafood (4)**	clams, mussels; squid, octopus; shellfish (cramps, shrimps, lobster); surimi and other fish-based food products
**Fruits (12)**	oranges; orange juice; bananas; apples or pears; peaches, nectarines, or apricots; watermelon or melon; grapes; prunes or plums; cherries, strawberries; kiwi; pineapple; olives
**Vegetables (9)**	spinach; cabbage, cauliflower or broccoli; lettuce or endive; tomatoes; onions; carrots or squash; eggplant, zucchini, or cucumber; green, red, or yellow peppers; and garlic
**Nuts (1)**	almonds, walnuts, peanuts and other types of nuts,
**Legumes (2)**	lentils, chickpeas, beans; peas, green beans
**Cereals & Pasta (4)**	breakfast cereals; corn; rice; pasta
**Bread (3)**	bread sticks; white and whole breads
**Potatoes (5)**	frozen French fry; homemade; boiled/stew; chips; other crisps
**Sweets & sugar (14)**	canned fruits; biscuits; chocolate biscuits; whole grain biscuits; cookies; baked goods; muffins; croissants and doughnuts;added sugar; candies; marmalade, honey; chocolate; peanut butter (eg. Nutella/Nocilla); chocolate/cocoa powder
**Vegetable Fat (4)**	olive oil; sunflower, corn oils; margarine; mayonnaise

### 24hour dietary recall (reference method)

Information on three non-consecutive 24hDR was collected from all children, including at least one non-weekday. Trained nutritionists collected information using the USDA Automated Multiple-Pass Method [30], to stimulate recall from parents on foods and beverages consumed by the child during the previous day, and to reduce potential the recall bias.

**Fig 1:**
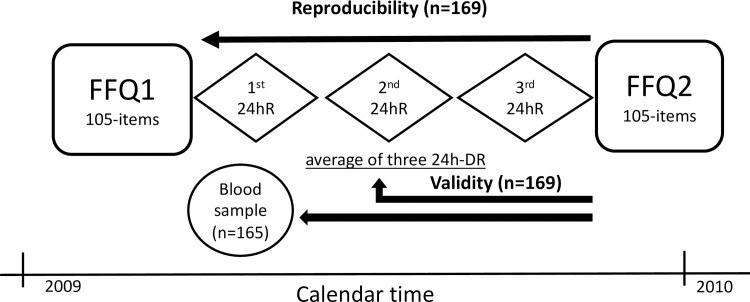
The design of the validation study among children aged 4–5 years of the INMA Project in Valencia, 2009–2010. FFQ, food frequency questionnaire; 24hDR, 24hour dietary recall.

The first 24hDR was completed in the same interview that the first FFQ was fulfilled; the second 24hDR was usually completed a few days or weeks later when a fasting blood sample was also taken for most children; and the third 24hDR was completed either by telephone interview or in personal interview in the middle or by the end of the 9-month period close to when the second FFQ was completed (Fig 1). Portions and servings size were carefully estimated by using household measures and detailed descriptions of the food, method of preparation and brands. One nutritionist performed the coding of all food items eaten in units of weight and volume as collected from the 24hDR in order to obtain dietary intakes using the Food Processor II® software. This software primarily uses food composition tables from the US Department of Agriculture although we added specific Spanish foods as published in food composition tables in Spain [27–29]. The average of all three 24hDR was used as a representation of individual intake (reference method)

### Biomarkers

Fasting blood samples were obtained from each child when they completed either the first or second 24hDR. Samples were collected during a whole year period between January 2009 and February 2010. A thorough protocol was designed to collect, transport and measure the blood samples for vitamin C, E, retinol and carotenoids, similarly to that used in previous studies [21, 31]. Briefly, blood samples were collected at clinical examination and properly stored in insulated dry containers at 4°C. Within 30 minutes of collection, the whole-blood samples were centrifuged at 6000 rpm for five minutes to separate blood cells and serum samples. The blood samples for vitamin C determination, wrapped in tin foil from collection, were mixed with equal volumes of freshly prepared metaphosphoric acid (10%) and transferred to screw-capped Eppendorf tubes to avoid vitamin C degradation. Samples were stored (- 80° C). Blood samples packed in dry ice were shipped to the central laboratory by dedicated couriers. Samples were filtered through 0.45 mm membranes (Type HA, Millipore, USA) and injected onto the HPLC. Chromatographic analysis was performed using a C18 μBondapak column (Waters, USA) and eluted with methanol (50%) and water (50%) containing acetic acid buffer and N-cetil-trimetil-ammonium bromide at a flow rate of 1 ml/min. Detection was performed by photodiode array detector set at 254 nm. Between-day variability was < 8% and quality control was contrasted by participating in the Vitamin C Quality Assurance Programme conducted by NIST (MD, USA). Serum concentrations of lutein/zeaxanthin, β-Crytoxanthin, lycopene, a-carotene and b-carotene in addition to vitamin A (retinol) and E (a- and g-tocopherol) were simultaneously measured by ultra-fast-liquid chromatography [32]. The short and long-term precision and accuracy of the analytical method of laboratory is verified periodically through participation in the Fat-Soluble Quality Assurance Programme conducted by the National Institute of Standards and Technology (NIST; Gaithersburg, MD, USA).

Plasma cholesterol, which was measured to adjust carotenoid concentrations, and other serum biochemical and hematologic parameters were determined at the General Biochemistry and Hematology Laboratories of the Hospital Puerta de Hierro, Madrid, according to routine quality-controlled standard methods.

### Statistical analysis

Statistical analyses were conducted with the R statistical software version 3.0.0 (R Foundation for Statistical Computing, Vienna, Austria; http://www.r-project.org). We calculated means and standard deviations for total nutrient intakes and food consumption from the FFQ, from the average of three 24hDR and from the biomarkers. We used paired Student’s test for means comparison of the individual daily nutrient intakes and food consumption reported in the different periods.

All nutrient and food group intakes were log-transformed prior to analysis to improve their normality. Energy-adjusted intakes were computed using the residual method, where each nutrient is regressed on total calories, and the population mean was then added back to the calculated residuals [22].

Since most carotenoids are transported in plasma lipoproteins, plasma concentrations of carotenoids and vitamin E were also adjusted per plasma cholesterol concentrations also using the residual method.

To assess the reproducibility of the FFQ, we estimated Pearson correlations coefficients to compare the individual energy-adjusted dietary intakes of nutrients and foods reported from the two FFQ completed at baseline and the end of the study period (Fig 1). We explored validity of the FFQ by calculating the Pearson correlation coefficients between individual energy-adjusted dietary intakes from the average of two FFQ (FFQav) and the average of three 24hDR nutrients intakes. Because day-to-day variance (within variability) tends to attenuate the correlation between FFQ and 24hDR, de-attenuated Pearson´s correlation coefficients were calculated by adding the factor √ 1+ {(S2w / S2b)/3} (24hDR was repeated 3 times) to the calculated Pearson´s correlation coefficient. In the formula, (S2w) represents within-person variance and (S2b) between-person variance for each nutrient. Pearson correlation coefficients were also used to evaluate the validity of the FFQ by comparing individual energy-adjusted dietary intakes from the first FFQ and their respective plasma concentrations of the nutrients vitamin C, E, retinol and carotenoids (α- carotene, β- carotene, lutein + zeaxanthin, lycopene and β-cryptoxanthin). Spearman correlation coefficients were also estimated although the results were very similar to those observed for parametric correlations. Therefore, only Pearson correlations are presented.

## Results

The main characteristics of the 169 children who participated in the validation study are presented in Table 2. Participants in the validation study had a mean age of 4.34 years, slightly higher than non-participant children, 4.30 years (p = 0,006). No other statistically significant differences were observed for other personal characteristics or BMI variables.

**Table 2:** Characteristics of children (n = 590) of the INMA-Valencia study according to participation in the validation study (Yes/No), 2009–2010.

	Participation in the validation study		
	YES (n = 169)	NO (n = 421)	p-value^2^
	Mean (SD)	Mean (SD)		
**Age (Years)**	4.34 (0.1)	4.30 (0.2)	0.006	
**Body Mass Index (Kg/m**^**2**^**)**	16.0 (1.5)	16.3 (1.8)	0.144	
	% (n)	% (n)		
**Body Mass Index (Kg/m**^**2**^**), in categories**^**1**^			0.733	
ormal	80 (137)	79 (331)		
Overweight—Obesity	20 (32)	21 (86)		
**Gender**			0.202	
Male	49 (82)	54 (227)		
Female	51 (87)	46 (190)		
**Socioeconomic status**			0.489	
I+II (high)	21 (36)	18 (73)		
III	26 (44)	25 (105)		
IV+V (low)	53 (89)	57 (239)		
**Education level**			0.452	
> = Primary School	33 (55)	27 (114)		
Secondary School	41 (70)	45 (186)		
University	26 (44)	28 (117)		
**Lunchroom**			0.843	
< 1 time/ week	30 (50)	31 (129)		
> = 1 time / week	70 (119)	69 (290)		
**Country of origin**			0.160	
Spain	93 (158)	90 (374)		
Other Countries	7 (11)	10 (43)		
	Mean (SD)	Mean (SD)		
**Energy (Kcals/day)**	1592 (328)	1569 (368)	0.490	
**Carbohydrates (g/day)**	195 (47.1)	193 (51.8)	0.732	
**Proteins (g/day)**	68.8 (14.2)	68.4 (15.3)	0.798	
**Fats (g/day)**	63.0 (15.3)	61.0 (16.7)	0.187	

^1^ Body Mass Index (kg/m2) categories according to age criteria by Cole et al, 2000

^2^ p-values from t Student-test (continuous variables) and from Chi-square tests (categorical variables)

The energy and macronutrients were very similar between participants and non-participants at baseline. No differences in mean daily energy and macronutrient intakes estimated by the first FFQ were observed between participants and nonparticipants children in the validation study (Table 2).

### Reproducibility

Table 3 presents the mean daily nutrient intakes and Pearson correlation coefficients between nutrient intakes estimated by the two FFQ completed in approximately a 9-month period. Except for total carbohydrates and alfa-carotene, estimates of energy and nutrients intakes were slightly lower in the first FFQ. Highly significant correlations were observed for most log-transformed nutrients, ranging from r = 0.30 for monounsatured fatty acids (MUFA) to r = 0.64 for beta-cryptoxanthin. The average of correlation coefficients was 0.41. When nutrient intakes were adjusted for total energy, correlation coefficients varied for some nutrients although in general, they were very similar; the average of coefficient correlations was 0.42.

**Table 3:** Mean daily nutrient intakes and Pearson Correlation coefficients among children (n = 169) of the INMA- Valencia study, 2009–2010.

	FFQ1^1^	FFQ2^1^	p-value^2^	Pearson coefficient correlations between FFQ1 and FFQ2		Percent of agreement ^5^
Nutrient intakes (units/day)	Mean (SD)	Mean (SD)		*r*^3^	*r* adj.^4^	
**Energy (kcals/day)**	1592 (328)	1628 (500)	0.437	0.35		68.0
**Protein (g/day)**	69 (14)	73 (23)	0.071	0.36	0.45	63.3
**Total carbohydrates (g/day)**	195 (47)	193 (65)	0.720	0.36	0.28	68.6
**Dietary fibre (g/day)**	15 (4.4)	15 (6.6)	0.914	0.35	0.30	61.5
**Cholesterol**	209 (49)	223 (87)	0.056	0.44	0.39	71.6
**Total fat (g/ day)**	63 (15)	66 (24)	0.121	0.33	0.31	68.0
**SFA (g/ day)**	23 (5.7)	23 (8.3)	0.404	0.39	0.46	70.4
**MUFA (g/day)**	26 (7.6)	28 (11)	0.110	0.30	0.30	66.9
**PUFA (g/ day)**	9.5 (2.6)	10 (4.6)	0.037	0.32	0.31	64.5
**Omega 3 (g/day)**	1.0 (0.3)	1.1 (0.4)	0.010	0.42	0.44	67.5
**Omega 6 (g/day)**	8.4 (2.4)	9.2 (4.3)	0.044	0.32	0.33	64.5
**Trans fatty acid (g/day)**	1.2 (0.4)	1.1 (0.5)	0.105	0.37	0.44	67.5
**Retinol (μg/day)**	498 (332)	556 (515)	0.222	0.34	0.36	71.6
**α-carotene (μg/day)**	296 (246)	265 (249)	0.246	0.39	0.38	71.0
**β-carotene (μg/day)**	1364 (866)	1454 (118)	0.427	0.53	0.53	75.1
**β-Cryptoxanthin (μg/day)**	170 (130)	202 (194)	0.080	0.64	0.62	74.0
**Lutein+Zeaxanthin (μg/day)**	935 (565)	1014 (734)	0.270	0.52	0.50	70.4
**Lycopene (μg/day)**	1466 (910)	2319 (1428)	<0.001	0.50	0.51	74.0
**Vitamin B6 (mg /day)**	1.3 (0.5)	1.4 (0.7)	0.006	0.42	0.31	68.0
**Folates (μg/day)**	185 (62)	199 (86)	0.100	0.43	0.49	65.7
**Vitamin B12 (μg/day)**	6.0 (2.4)	6.8 (4.1)	0.025	0.41	0.33	68.6
**Vitamin C (mg /day)**	64 (46)	79 (80)	0.029	0.60	0.56	71.0
**Vitamin D (μg/day)**	4.2 (2.4)	4.6 (2.3)	0.141	0.39	0.45	68.6
**Vitamin E (mg/day)**	8.0 (3.4)	8.9 (4.6)	0.015	0.40	0.52	65.1
**Calcium (mg/day)**	1048 (306)	1059 (419)	0.789	0.39	0.53	72.2
**Iron (mg/day)**	10.4 (4.8)	10.7 (7.0)	0.684	0.43	0.43	65.1
**Magnesium (mg/day)**	235 (55)	242 (80)	0.339	0.38	0.53	68.6
**Sodium (mg/day)**	2131 (469)	2195 (733)	0.340	0.33	0.30	64.5
**Zinc (mg/day)**	8.6 (2.0)	9.1 (3.0)	0.071	0.40	0.43	66.9
**Iodine (μg/day)**	144 (48)	145 (61)	0.827	0.42	0.54	69.8
***Average of correlation coefficients ***				**0.41**	**0.42**	**68.5**

^1^ FFQ1 & FFQ2, the same FFQ was firstly administered at baseline (FFQ1) and secondly (FFQ2), between 6 to 9 months later

^2^ P-value from paired t-tests

^3^coefficient correlations after nutrient crude intakes were log-transformed

^4^correlation coefficient using nutrient intakes adjusted for total energy

^5^ Overall percentage of children classified in the same or an adjacent quintile; SFA, saturated fatty acids; MUFA, monounsaturated fatty acids; PUFA, Polyunsaturated fatty acids; all correlation coefficients were statistically significant, p<0.01.

According to classification into quintiles of nutrient intakes as estimated by the two FFQ, the percentage of agreement (i.e. children classified in the same or adjacent quintile) ranged between 61.5% for dietary fiber and 75.1% for beta-carotene (Table 3); the average of percentage agreement for nutrient intakes was 68.5%.

Regarding the reproducibility of the FFQ for food group intake, Table 4 shows the mean daily intake for the 17 foods and food groups estimated by the two FFQ. Meat, fish and fruit intakes estimates by the second FFQ were significantly higher than those estimated by the first (p<0.05). The correlation coefficients of food group intakes between the two FFQ showed a wider range of variation than that observed for nutrients, ranging from r = 0.16 for white meat to r = 0.72 for dairy products, although the average of correlation coefficients was slightly higher than that observed for nutrients, r = 0.43 (r = 0.44 for the energy-adjusted intakes) and also for percentage of agreement (71%).

**Table 4:** Mean daily food and food groups intake and Pearson Correlation coefficients among children (n = 169) of the INMA–Valencia study, 2009–2010.

	FFQ1^1^	FFQ2^1^	p^2^	Pearson coefficient correlations between FFQ1 and FFQ2		Percent of agreement ^5^
Food Groups intake (g/day)	Mean (SD)	Mean (SD)		*r*^3^	*r* adj.^4^	
**Dairy Products (g/day)**	541 (215)	539 (272)	0.940	0.72	0.80	74.6
**Eggs (g/day)**	14 (5)	13 (7)	0.332	0.39	0.40	99.4
**White meat (g/day)**	24 (11)	27 (15)	0.085	0.16^a^	0.18 ^a^	63.3
**Red meat (g/day)**	25 (13)	30 (21)	0.005	0.40	0.38	66.3
**Processed meat (g/day)**	31 (12)	32 (22)	0.596	0.41	0.39	66.9
**Fatty fish (g/day)**	8 (7)	13 (12)	<0.001	0.35	0.34	62.1
**Lean fish (g/day)**	15 (10)	18 (11)	0.045	0.41	0.43	63.3
**Seafood (g/day)**	8 (5)	11 (9)	0.002	0.56	0.50	71.6
**Fruits (g/day)**	155 (123)	203 (252)	0.027	0.54	0.51	72.2
**Vegetables (g/day)**	58 (41)	57 (43)	0.811	0.64	0.65	78.1
**Nuts (g/day)**	4 (4)	4 (10)	0.874	0.40	0.41	86.4
**Legumes (g/day)**	18 (11)	21 (20)	0.094	0.39	0.41	66.9
**Cereals and Pasta (g/day)**	59 (26)	63 (30)	0.156	0.24	0.27	68.6
**Bread (g/day)**	73 (39)	60 (46)	0.007	0.34	0.36	63.9
**Potatoes (g/day)**	32 (15)	29 (16)	0.125	0.58	0.60	64.5
**Sweets and sugar (g/day)**	55 (29)	49 (37)	0.072	0.43	0.49	72.8
**Vegetable Fat (g/day)**	11 (8)	13 (11)	0.080	0.34	0.35	66.3
***Average of correlation coefficients ***					**0.43**	**0.44**	**71.0**

^1^FFQ1 & FFQ2, the same FFQ was firstly administered at baseline (FFQ1) and secondly (FFQ2), between 6 to 9 months later

^2^ P-value from paired t-test

^3^coefficient correlations after nutrient crude intakes were log-transformed

^4^correlation coefficient using nutrient intakes adjusted for total energy

^5^ Overall percentage of children classified in the same or an adjacent quintile

^a^p<0.05, all other coefficients p<0.01.

### Validity

Table 5 presents coefficient correlation for the validity of the FFQ by comparing the mean daily energy and nutrient intakes based on the average of the two FFQ and the average of the three 24hDR. Intakes from the FFQs were on average 15–20% higher than that estimated by the three 24hDR (p<0.001). In general, significant correlation coefficients were observed for most nutrients, ranging from r = 0.05 for omega3 fatty acid intake to r = 0.54 for calcium intake; the average of correlation coefficients was 0.30. When the analysis was based on energy adjusted nutrient intakes, the magnitude of correlation coefficients and the average were very similar to the correlations with unadjusted estimates. The de-attenuated Pearson correlation coefficients ranged from r = 0.19 (PUFA) to r = 0.91 (iron), and the average of de-attenuated Pearson correlation coefficients was r = 0.44.

**Table 5:** Comparison between mean daily nutrient intakes from the average of two FFQs and three24hDRs and correlation coefficients among children (n = 169) of the INMA–Valencia study, 2009–2010.

	FFQav^1^	24hDRav^2^	P^3^	Pearson coefficient correlations between FFQav and 24hDRav			Percent of agreement ^7^
Nutrients (units/day)	Mean (SD)	Mean (SD)		*r*^4^	*r* adj.^5^	*r* de-att.^6^	
**Energy (kcals/day)**	1610 (350)	1255 (183)	<0.001	0.29			64.5
**Protein (g/day)**	70 (16)	53 (9.5)	<0.001	0.30	0.38	0.55	62.7
**Total carbohydrates (g/day)**	194 (48)	161 (29)	<0.001	0.30	0.29	0.41	60.4
**Dietary fiber (g/day)**	15 (4.7)	11 (3.5)	<0.001	0.26	0.22	0.32	63.9
**Cholesterol**	216 (58)	178 (61)	<0.001	0.31	0.21	0.30	62.7
**Total fat (g/ day)**	65 (16)	46 (8.9)	<0.001	0.26	0.18	0.26	60.9
**SFA (g/ day)**	23 (5.9)	18 (4.0)	<0.001	0.39	0.40	0.58	63.3
**MUFA (g/day)**	27 (7.5)	19 (4.0)	<0.001	0.21	0.14	0.20	62.7
**PUFA (g/ day)**	9.9 (2.9)	5.8 (1.4)	<0.001	0.11	0.13	0.19	57.4
**Omega 3 (g/day)**	1.0 (0.3)	0.8 (0.3)	<0.001	0.05	0.19	0.27	52.7
**Omega 6 (g/day)**	8.8 (2.7)	4.9 (1.3)	<0.001	0.15	0.16	0.23	57.4
**Trans fatty acids (g/day)**	1.1 (0.4)	0.5 (0.4)	<0.001	0.21	0.17	0.23	55.6
**Retinol (μg/day)**	527 (342)	291 (148)	<0.001	0.26	0.21	0.30	57.4
**α- carotene (μg/day)**	280 (213)	204 (234)	0.002	0.31	0.34	0.49	66.3
**β- carotene (μg/day)**	1409 (888)	681 (600)	<0.001	0.34	0.38	0.55	60.9
**β- Cryptoxanthin (μg/day)**	186 (147)	69 (108)	<0.001	0.30	0.31	0.44	62.7
**Lutein+Zeaxanthin (μg/day)**	974 (572)	364 (465)	<0.001	0.25	0.30	0.43	63.3
**Lycopene (μg/day)**	1893 (1003)	702 (1038)	<0.001	0.36	0.37	0.53	62.1
**Vitamin B6 (mg /day)**	1.4 (0.5)	1.0 (0.4)	<0.001	0.29	0.32	0.46	63.9
**Folato (μg/day)**	192 (64)	124 (38)	<0.001	0.23	0.23	0.33	55.0
**Vitamin B12 (μg/day)**	6.4 (2.7)	3.7 (1.6)	<0.001	0.29	0.32	0.46	62.1
**Vitamin C (mg /day)**	72 (59)	38 (24)	<0.001	0.36	0.40	0.57	62.1
**Vitamin D (μg/day)**	4.4 (2.0)	4.2 (1.7)	0.348	0.36	0.29	0.42	62.1
**Vitamin E (mg/day)**	8.3 (3.3)	3.7 (1.0)	<0.001	0.25	0.22	0.31	56.8
**Calcium (mg/day)**	1054 (309)	784 (205)	<0.001	0.54	0.58	0.84	70.4
**Iron (mg/day)**	10.5 (4.9)	8.8 (4.6)	<0.001	0.49	0.63	0.91	70.4
**Magnesium (mg/day)**	238 (58)	178 (33)	<0.001	0.36	0.33	0.48	61.5
**Sodium (mg/day)**	2163 (498)	1689 (440)	<0.001	0.19	0.25	0.36	57.4
**Zinc (mg/day)**	8.9 (2.2)	6.4 (1.2)	<0.001	0.32	0.37	0.53	59.8
**Iodine (μg/day)**	145 (47)	92 (38)	<0.001	0.46	0.34	0.49	65.7
***Average of correlation coefficients***				***0*.*30***	***0*.*31***	***0*.*44***	***61*.*7***

^1^ Average of FFQ1 and FFQ2

^2^Average of the three 24hDR

^3^p-value from t-tests

^4^ coefficient correlations after nutrient intakes were log-transformed

^5^ correlation coefficient using energy-adjusted nutrient intakes

^6^ de-attenuated correlation coefficients after nutrient intakes were log-transformed and energy-adjusted

^7^ Overall percentage of children classified in the same or an adjacent quintile; SFA, saturated fatty acids; MUFA, monounsaturated fatty acids; PUFA, Polyunsaturated fatty acids; *r*≥0.2, p-value <0.01; 0.15≤ *r*≤0.19, p value <0.05; *r*<0.15, p-value >0.05.

Regarding the biochemical validity of the FFQ, Table 6 shows the comparison between the mean daily intake for ten nutrients measured by the first FFQ and the mean plasma concentration for those nutrients; it also presents the intake of fruits and vegetables from the first FFQ in comparison with mean plasma concentration for total carotenoids. For log-transformed intakes, the correlation coefficients between FFQ and plasma concentration (biochemical validity) were lower for lutein+zeaxanthin (r = 0.09), β-carotene (r = 0.15), alfa-carotene (r = 0.12) and retinol (r = 0.15) and higher for vitamin C (r = 0.33), β-cryptoxanthin (r = 0.37) and vitamin E (r = 0.23). Overall, the average of correlation coefficient was r = 0.21. When the analysis was based on energy-adjusted intakes correlations tended to improve and the average of correlation coefficients was r = 0.22. The correlation coefficient between fruit and vegetable intake and plasma concentration of carotenoids was r = 0.14. Between 55.8% (retinol) and 64.8% (beta-cryptoxantine) of children were classified in the same or adjacent quintiles by the FFQ vs quintiles of plasma concentration of plasma nutrients.

**Table 6:** Mean daily nutrient and food intakes by the first FFQ and nutrient plasma concentration and Pearson correlation coefficients in children (n = 165) of the INMA–Valencia study, 2009–2010.

	FFQ1	Plasma Concentration	Pearson coefficient correlations between FFQ1 and plasma concentrations		Agreement (%) ^3^
Nutrients and foods	Mean (SD)	Mean (SD)	*r*^1^	*r*^2^	
**Vitamin E (μg/dl)**	7.9 (3.4)	1105 (178)	0.23 ^a^	0.29 ^a^	61.2
**Lutein + Zeaxanthin (μmol/dl)**	942 (568)	19 (4.7)	0.09	0.05	57.6
**β- Cryptoxanthin (μmol/dl)**	169 (131)	23 (13)	0.37 ^a^	0.40 ^a^	64.8
**Lycopene (μmol/dl)**	1478 (912)	53 (20)	0.27 ^a^	0.31 ^a^	56.4
**α- carotene (μmol/dl)**	297 (248)	19 (6.5)	0.12	0.12	57.0
**β- carotene (μmol/dl)**	1367 (869)	34 (16)	0.15	0.15	60.6
**Retinol (μmol/dl)**	505 (333)	31 (6.2)	0.14	0.06	55.8
**Vitamin C (μmol/l)**^**4**^	64 (47)	55 (18)	0.33 ^a^	0.35 ^a^	61.1
			**0.21**	**0.22**	**59.3**
**Fruits and vegetables (g/day) vs. total carotenoids (μmol/dl)**^**5**^	213 (143)	95 (29)	0.14	0.14	58.2

^1^ Coefficient correlations after nutrient intakes were log-transformed

^2^correlation coefficient using energy-adjusted nutrient intakes and cholesterol adjusted for carotenoids and vitamin E

^3^ Overall percentage of children classified in the same or an adjacent quintile;

^4^ n = 157

^5^ Carotenoids sum of α- carotene, β- carotene, Lutein + Zeaxanthin, β- Cryptoxanthin, Lycopene;

^a^ p<0.01, all other p>0.05

## Discussion

The results of this study support that the FFQ is an adequate method for dietary assessment among children aged 4 to 5 years of the INMA study. The correlation coefficients of reproducibility for the FFQ ranged from 0.3 to 0.7 for most nutrients and foods groups which may be considered in general as a moderate reproducibility. In addition, the correlations for validity of the FFQ when compared with three 24hDR and the plasma concentration of several nutrients (carotenoids, vitamins C, E and retinol) was in general higher than 0.20 which may be considered as a low-moderate validity. To our knowledge, this is the first validation study of a FFQ carried out in a population of children aged 4 to 5 years in Spain with a sample size enough to detect statistically significant correlations of interest for validity (r>0.20).

The correlation coefficients for most of the nutrients and foods groups were comparable with those observed in other validation studies of FFQ in children and young populations [10, 11, 31–36]. In our study, reproducibility was assessed by comparing the results from the FFQ administered twice over a period of 9 months, while validity was examined by comparing the nutrient and food intakes from FFQ with the intakes from three 24hDR and several nutrient intakes in plasma (carotenoids, vitamins C, E and retinol). Given the general characteristics of the study population, 24hDR were considered a more feasible reference method than others such as food diaries since they are less demanding and cumbersome for participants. The use of both reference methods to evaluate the validity of the FFQ was considered appropriate since they have different sources of error from the FFQ and correlation is unlikely [16, 37].

Few studies have published data of reproducibility of FFQ in children [13–15, 38–41] and only one, carried out in forty-seven Japanese children aged 6 years, used a period of six months to examine reproducibility, closer to the period used in our study [41]. The average of reproducibility coefficients in our study was 0.42, lower than that observed in the Japanese study that reported correlations higher than 0.50 for all nutrients. In an Australian study with 101 children aged 9 to 16 years, the correlation coefficients of reproducibility assessed in a five-month period ranged from 0.18 (vitamin A) to 0.50 (calcium) and the average of correlation coefficients was 0.32 [39]. In a study carried out among 94 elementary school children in Puerto Rico, reproducibility correlations were much lower, probably related to the low capability of children to remember their diet and despite the fact that FFQ were administered within a 2-week separation period only [41]. In two recently published studies [13–14] the reported correlations for reproducibility were slightly higher than in our study although the two FFQ were administered within a very short period (one month).

The mean daily intakes estimated for most nutrients and foods were higher in the second FFQ than in the first although, in general, the differences were small and significant only for a few nutrients (e.g., lycopene, fatty acids) and foods (eg. read meat, fatty fish, seafood and fruits); this may support that there were no major changes in children´s diet during the study period. While some studies had reported higher intakes in the first FFQ [38], in general, most studies have shown no evidence of major changes during the period of reproducibility [35].

Regarding reproducibility for food intakes, a study among 51 four to five grade students in US reported correlation coefficients for one-year reproducibility of a FFQ from -0.26 for vegetables and 0.40 for fruit juice [15], whereas our study showed correlation coefficients ranging from 0.05 for white meat to 0.73 for dairy products. Other studies reported higher values for reproducibility coefficients, although the period used to administer the two FFQ was shorter, from 2 to 4 weeks, which might artificially cause higher correlations [8, 14]. Overall, our FFQ showed a satisfactory reproducibility for most nutrients and food groups, particularly for those more frequently eaten and, therefore, it may be considered a reliable dietary assessment method in children aged 4–5 years of the INMA Project, and probably, in children of the same age in Spain since this was a population based study.

Regarding the validity of the FFQ, we used the average of the two FFQ to get more stable estimates, and compared the results against the two reference methods used. An average of three 24hDR has been considered adequate to estimate energy and some nutrients [42]; a higher number of recalls was not feasible in our study due to limited resources and, on the other hand, a higher number of recalls may increase the risk of fatigue, boredom and training effects [43]. The validity coefficient for energy intake between the FFQs and the average of three 24hDR in our study was 0.29 higher than in a study with 68 pre-school children in USA (r = 0.08) that also used 24hDR as the reference method [11]; correlations for other nutrients in that study ranged from 0.27 for alpha-tocopherol to 0.42 for vitamin C, similar to those found in our study for energy-adjusted nutrients. In another study among children aged 9 to 18 in USA (n = 261), validity was evaluated against three 24hDR during a one year period [44]; the reported average of correlation coefficient after correction for within-person error was higher than in our study (0.51 vs 0.44) and the observed in another study among schoolchildren aged 6–9 years from Brazilian Western Amazon (0.46) [45], which could be due in part to the higher capacity of older children to remember their diets.

In our study the mean nutrients and energy intake derived from the three 24hDR were, in general, about 15–20% smaller than those estimated by the two FFQ; similar differences have been also reported in most studies [10, 11, 15, 46]. Even when administrated by trained interviewers, 24hDR may underestimate nutrient intakes against FFQ although the main interest in validation studies is to evaluate the FFQ capacity to classify participants instead of obtaining absolute nutrient estimates [5]. A possible explanation for the lower estimates by the 24hDR may be that 71% of children were school meal users and parents or care-givers did not know accurately the food intake of the children even though they were told to ask for that information. It may also be possible that telephone interviews for the second 24hDR might have contributed to some underestimation. On the other hand, it has been suggested that overestimation may also occur when FFQ with many food items are used [4] although our 105-item FFQ was similar to others used in literature and took some 30 minutes to complete. This might also explain why some Pearson coefficients for validity against recalls were low (e.g., PUFA, omega3 and trans fatty acids); in fact, when correlation coefficients were estimated in children who did not have school meals (n = 50), the average of correlation coefficients improved r = 0.34 (data not shown).

Regarding the relative validity of the FFQ assessed by biomarkers, poor correlations were found between dietary intake and plasma concentrations of retinol, α-carotene and lutein+zeaxanthin. Low validity coefficients have been also found in other studies with children [8] as well as in other populations [5, 31]. Although serum nutrient concentrations provide an independent measure of nutrient intake, many are influenced by nondietary factors. Retinol concentrations in plasma is highly regulated by liver stores over a wide range of dietary intakes that can be found mainly in subjects with either severely depleted or highly saturated liver stores [47]. For vitamin E, it has been suggested that plasma concentration may not be a good marker for usual nutrient intake among children and that the adipose tissue may better represent usual vitamin E intake [37]. Nevertheless, the lack of validity for retinol deserves attention when using this FFQ in the study of diet disease. Our results support that lycopene and β-cryptoxanthin may be more sensitive to dietary intake and would be more appropriate markers for nutritional intake. In general, validity coefficients found for biomarkers in our study have been similar or slightly lower to those found in other studies in children at older or younger ages [11, 34].

Correlations between FFQ and biomarkers estimates may differ among studies due to the bioavailability of nutrients in the food, the use of different data bases, and other factors such as fat content of diet or nutrient absorption [48]. It has been reported that some adults with higher fat mass may accumulate a larger proportion of ingested carotenoids in the adipose tissue and a smaller one in circulation [31, 34]. Thus, when the correlation analysis was stratified by weight status, correlations for lutein+zeazanthin and β-cryptoxantin were higher in overweight and obese children; on the other hand, correlations for vitamin E and lycopene were higher in normal weight children (data not shown).

Another factor that may influence the correlations between FFQ and blood concentration of vitamins is the vitamin content of foods according to the season of the year and, consequently, when blood samples were collected. Except for lutein+zeaxanthin and vitamin C, all correlation coefficients were higher in children whose blood samples were collected in autumn (data not shown).

A potential limitation of our study is that parents and children´s care-givers answered the questionnaires. This fact could affect the reproducibility and validity of the FFQ because consciously or unconsciously care-givers may report the desired diet for their children and moreover, they may not know what their children eat when they are not in their charge. Trained interviewers carefully administered both FFQ and three 24hDR in personal interviews mostly to mothers, asking them to complete missing information in those children who ate at school canteens, or with their grandparents or car-givers. Another limitation might be the use of a 9-month period to evaluate the validity and the reproducibility of the questionnaire which could lead to an underestimation of certain nutrients that depend on the season of the year. However, the use of a longer period (e.g one year) might be inappropriate in children as their food intake may change rapidly and increase the risk of poor performance for questionnaire reproducibility and validity [38]. We must also consider that both dietary methods used, the FFQ and 24hDR, depend on memory, that the use of 24hDR can present certain disadvantages as a gold standard, particularly if they are telephone based, and that the measurement errors of the two methods may be not completely independent. However, the two methods rely on different kinds of memory; the FFQ is related to generic memory whereas 24hDR relies on episodic memory, so that errors are unlikely to be related. On the other hand, 24hDR were conducted by trained interviewers (nutritionists) who employed strategies to ensure that foods were not forgotten and checked questionable responses to obtain accurate estimation of real food intake.

In conclusion, our findings show that both reproducibility and validity of the FFQ assessed in this study in children aged 4–5 years in Spain using three 24hDR and biological markers was low to moderate for most intakes but comparable to other studies in children with lower sample size. Despite some low correlations, overall the results suggest that our FFQ is a good method for assessing usual intake and classifying children according to their dietary intakes.

## Supporting Information

S1 Fig Flow chart describing the selection process among participants of the validity and reproducibility of FFQ in the INMA-Valencia prospective study to be included in the present analyses (TIFF)

## Abbreviations

FFQ: food frequency questionnaire
24HR: 24-hour recalls
INMA: Project, Spanish Childhood and Environment Project
BMI: Body mass index
FFQav: the average of two food frequency questionnaire
S2w: within-person variance
S2b: between-person variance
MUFA: monounsatured fatty acids
PUFA: Polyunsaturated fatty acids
SFA: saturated fatty acids
